# Chemical Lecture Notes

**Published:** 1891-12

**Authors:** 


Chemical Lecture Notes. Taken from Prof. C. O. Curtman's Lectures at the St. Louis College of Pharmacy. By H. M. Whelpley, M. D., Ph. G., F. R. M. S., Professor of Microscopy and Quiz Master of Pharmacognosy and Botany in the St. Louis College of Pharmacy, etc. Third edition, with over one hundred illustrations. Pp. 211. Published by the author.

This is one of the most complete set of lecture notes we have seen for some time. It is an excellent work, replete with information given in a clear and concise manner. It is just the kind of a book for a quiz master, or for the busy professional man who wants to brush up his knowledge of chemistry, which has grown dim from lack of use. J. A. M.

